# Unresectable leiomyosarcoma of the inferior vena cava with right atrium tumor thrombus: when to deem this tumor inoperable? A case report and literature review

**DOI:** 10.3389/fonc.2023.1331896

**Published:** 2024-01-04

**Authors:** Luis D. Castellanos, Marina M. Tabbara, Alan S. Livingstone, Tomas A. Salerno, Javier Gonzalez, Gaetano Ciancio

**Affiliations:** ^1^ Department of Surgery, Leonard M. Miller School of Medicine, University of Miami, Miami, FL, United States; ^2^ Miami Transplant Institute, Jackson Health System, Miami, FL, United States; ^3^ Division of Surgical Oncology, Leonard M. Miller School of Medicine, University of Miami, Miami, FL, United States; ^4^ Division of Cardiothoracic Surgery, Leonard M. Miller School of Medicine, University of Miami, Miami, FL, United States; ^5^ Servicio de Urología, Hospital General Universitario Gregorio Marañón, Madrid, Spain; ^6^ Department of Urology, Leonard M. Miller School of Medicine, University of Miami, Miami, FL, United States

**Keywords:** leiomyosarcoma, inferior vena cava, primary retroperitoneal sarcoma, case report, cardiac tumor

## Abstract

Leiomyosarcomas (LMS) of the inferior vena cava (IVC) are a rare form of retroperitoneal malignancy, and their venous extension to the right atrium is an even rarer event. These tumors pose a unique surgical challenge and often require a multidisciplinary team-based approach for their surgical treatment. We present a case of a 68-year-old man with primary LMS of the IVC with a tumor thrombus extending into the right atrium that was initially deemed inoperable. After extensive neoadjuvant chemo-radiation with minimal tumor effect, the patient underwent en bloc surgical resection of the tumor along with removal of the infrarenal IVC and right kidney and adrenal without the need for cardiopulmonary bypass. This case demonstrates the successful management of a primary LMS of the IVC with right atrial extension using a multimodal approach of neoadjuvant chemo-radiation and en bloc surgical resection without cardiopulmonary bypass. This strategy may offer a curative option for selected patients with these rare and aggressive tumors, improving their survival and quality of life.

## Background

Leiomyosarcomas (LMS) are rare neoplasms arising from smooth muscle cells, mostly affecting the gastrointestinal tract, uterus, skin, and blood vessels (1–3). Primary vascular LMS (PVL) has been reported to extend throughout the IVC and into the right atrium (RA) in around 20% of cases (4). The prognosis is poor, with reported survival rates ranging from 31% to 62% (5). Historically, IVC LMS were considered inoperable and their responsiveness to cytotoxic chemotherapy was limited, but with improvements in surgical techniques and perioperative management, surgical resection is now feasible (6). Neoadjuvant chemotherapy and/or radiation have been shown to reduce tumor burden (5); new advances in this area include the use of doxorubicin and trabectedin, either alone or in combination therapy (7). Curative surgical resection remains the treatment of choice for primary LMS of the IVC; however, due to limited experience with this disease, the optimal management is unknown. Nonetheless, surgical resection following neoadjuvant therapy should be strongly considered.

## Case presentation

**Figure 1:**
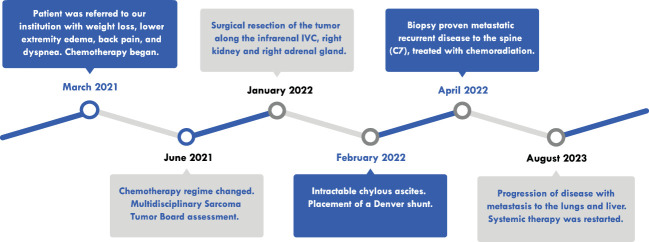
Timeline of the patient’s case.

**Figure 2:**
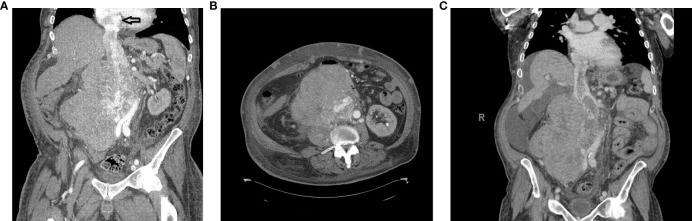
**(A)** Computed tomography (CT) scan coronal view showing a right retroperitoneal mass involving the inferior vena cava (IVC) and right kidney with tumor thrombus extending to the RA (black arrow). The hypervascular mass was 21.4 × 15.4 × 16.8 cm. **(B)** CT scan axial view of the IVC mass. **(C)** CT scan coronal view following chemoradiotherapy.

A 68-year-old man presented to another institution with weight loss, bilateral lower-extremity edema, lower back pain, and dyspnea on exertion (**Figure 1**). Contrast-enhanced computed tomography (CT) scan revealed a 16.1 × 14.6 × 20.7 cm mass in the right retroperitoneum involving and occluding the IVC with a tumor thrombus extending to the RA (**Figures 2A, B**). There were extensive venous collateralization, mass effect on the right kidney, and encasement of its ureter with associated hydronephrosis. A CT-guided needle core biopsy demonstrated intermediate grade 2/3 LMS. Due to thrombus extension into the retrohepatic IVC and RA, as well as adjacent organ involvement (uncinate process of the pancreas, infrarenal aorta), his disease was considered not safely resectable. It was recommended that he pursue systemic therapy as the first step in care.

The patient presented to our center to initiate chemotherapy. His BSA was 2.4 m^2^ at the time of treatment. He received in March 2021 five cycles of Adriamycin 75 mg/m^2^ and dacarbazine 900 mg/m^2^, and CT posttreatment revealed that the mass was stable. In June 2021, chemotherapy was changed to four cycles of gemcitabine 675 mg/m^2^ mg and docetaxel 75 mg/m^2^ mg with minimal response. At this point, the case was presented to a Multidisciplinary Sarcoma Tumor Board (MSTB) for discussion. The treatment plan was to combine 28 cycles of radiation therapy with a dose-escalated regimen of Pazopanib, up to a maximum of 600 mg daily. However, the patient did not tolerate this dose and was reduced to 400 mg. Upon completion, CT scan post-chemoradiotherapy showed a reduction in the size of the tumor (**Figure 2C**). Echocardiography revealed normal left ventricular systolic function with an ejection fraction of 67.4% and mildly increased right ventricular systolic pressure at 40–45 mmHg. The mass in the atrium was substantially smaller, being only 3.4 × 1.6 cm in size compared with 6 × 3 cm in size a couple of months earlier before radiation and Pazopanib therapy. Transesophageal echocardiography (TEE) showed that there was a multi-lobulated echo dense mass extending 2.2 cm into the RA from within the central lumen of the IVC. Still, it appeared to be somewhat mobile and did not appear to be adherent to the atrial walls or the intrapericardial and suprahepatic IVC.

In January 2022, the decision to proceed with surgical resection of the tumor with possible right nephrectomy, sternotomy, and cardiopulmonary bypass (CPB) *via* a multidisciplinary approach team of surgical oncologists, cardiovascular surgeons, and transplant surgeons was made. The risks and benefits of this operation were discussed with the patient, and signed informed consent was obtained.

## Procedure in detail

The surgical approach involved a midline incision that was further extended with a right subcostal incision for better exposure of the IVC. A Thompson retractor was placed. The tumor involved the right side of the retroperitoneum encompassing the right kidney and ureter. The right kidney was mobilized laterally and posteriorly, and the renal artery was ligated between the IVC and aorta and divided. Using piggyback techniques, the liver was mobilized off the IVC and the major hepatic veins and porta hepatis were isolated; this involved dissecting the ligamentum teres, followed by cautery division of the falciform ligament, right superior coronary, and triangular ligaments. The visceral peritoneum overlying the right hepatic hilum and subhepatic IVC were then incised together with the right inferior coronary and hepatorenal ligaments; afterward, the liver was rolled to the left abdomen. Surgical control of the hepatic hilum was performed, permitting isolation and control of the porta hepatis to permit a Pringle maneuver. Then, a piggyback maneuver can be performed. Minor hepatic veins draining into the anterior surface of the IVC were ligated and divided, allowing the subhepatic, retrohepatic, and suprahepatic portions of the IVC to be completely exposed. Finally, the posterior surface of the IVC was completely dissected in order to obtain total circumferential dissection of the IVC; the liver was dissected off the IVC, attached to it only by the major hepatic veins (8). The central diaphragm tendon was dissected to identify the intra-pericardial IVC. The right and left inferior phrenic veins were engorged due to obstruction of the IVC. The right inferior phrenic vein was stapled, and the left one was ligated at the time of complete occlusion of the IVC to avoid congestion of the liver.

**Figure 3:**
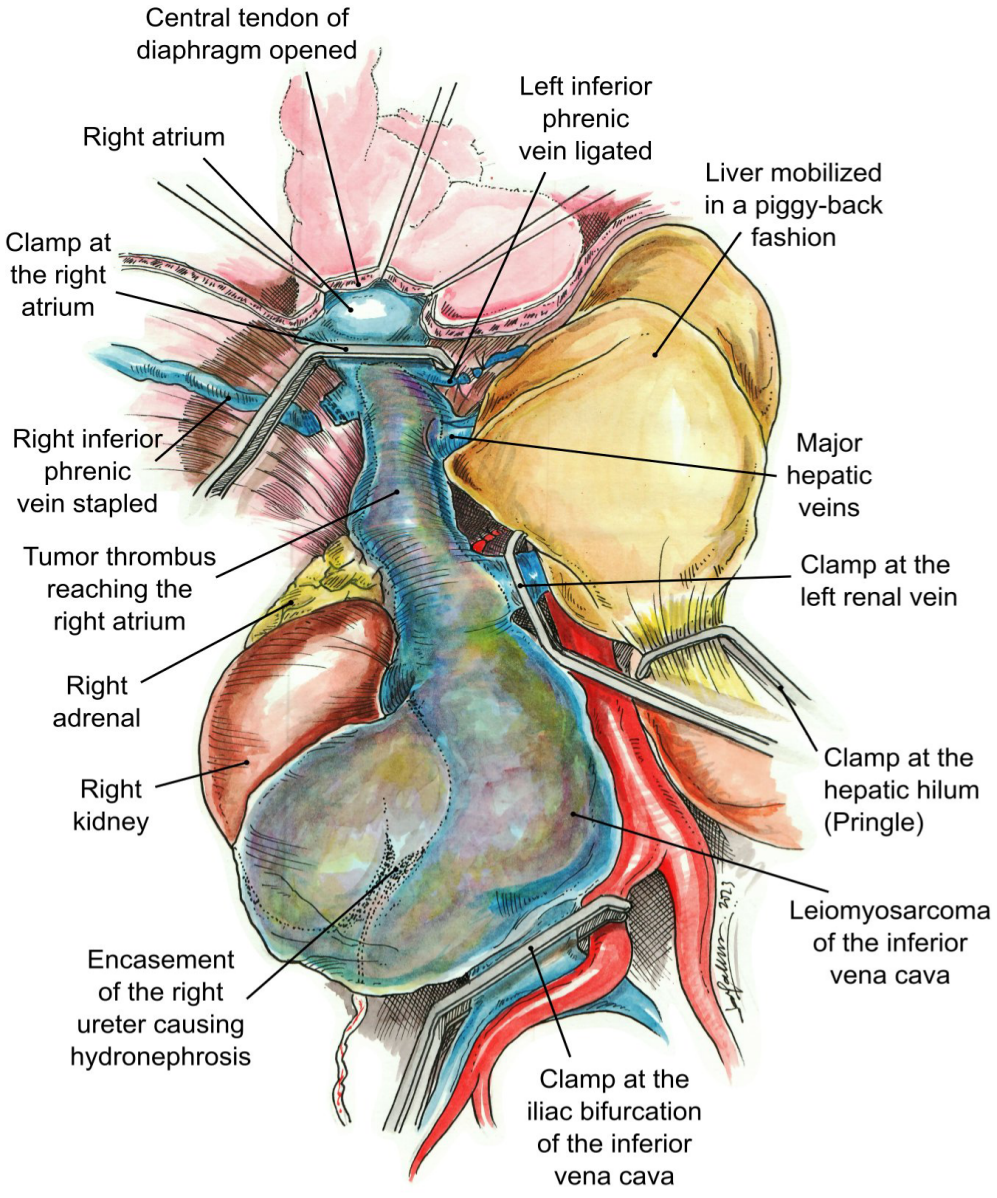
Vascular control. The clamps have been placed at the right atrium, iliac bifurcation of the inferior vena cava, left renal vein, and hepatic hilum (Pringle maneuver).

**Figure 4:**
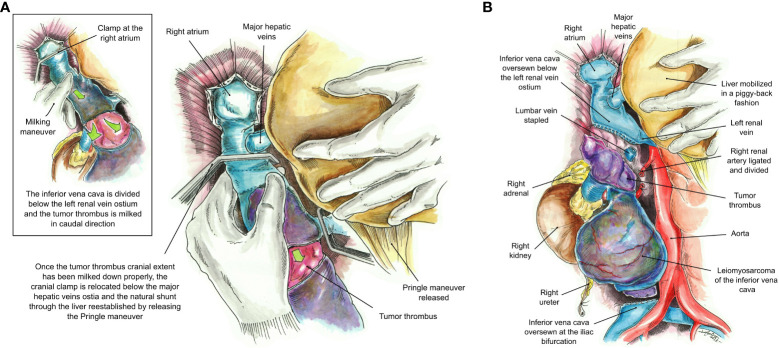
**(A)** Milking maneuver. The inferior vena cava is divided at a level below the left renal vein and the tumor thrombus is milked down to a level below the major hepatic veins’ ostia. Once the tumor thrombus has been completely milked down, the cranial clamp at the RA is relocated below the major hepatic veins’ ostia, and the natural shunt through the liver re-established by releasing the Pringle maneuver. **(B)** The inferior vena cava has been oversewn at a level below the left renal vein and the iliac bifurcation without the use of a vascular graft. The final specimen includes the leiomyosarcoma, right kidney and adrenal, and the entire tumor thrombus.

Intraoperative TEE was utilized to delineate the cranial extent and mobility of the tumor thrombus (TT). Vascular isolation of the IVC was achieved inferior and superior to the TT including both renal veins. Pringle maneuver was performed to occlude liver blood supply (**Figure 3**) temporarily. Vascular clamps were placed in the infra-renal vena cava just above the IVC bifurcation and the left renal vein. Under TEE monitoring, a vascular clamp was placed across the RA above the junction of the RA and intra-pericardial IVC. The cava was opened below the left renal vein. The TT was pulled and removed from the IVC. The vascular clamp was repositioned below the major hepatic veins. The Pringle maneuver lasted 5 min and was discontinued, reestablishing hepatic blood flow. The remaining IVC below the hepatic veins was sutured including only the left renal vein. All the lumbar veins were stapled due to their size. The infrarenal IVC was removed en bloc along with the right kidney and right adrenal gland (**Figure 4**) without requiring CPB or the use of a vascular graft. The TEE ensured that there were no pulmonary artery emboli perioperatively.

Blood loss and packed red blood cell (PRBC) transfusions were 2,000 cc and 13U of PRBC, respectively. Final pathology revealed a high-grade 3/3 LMS that measured 26 × 20 × 13 cm. The right kidney and adrenal gland were free of tumors. Five lymph nodes and all margins were negative for malignancy.

In the postoperative period, the patient developed significant chylous ascites intractable to medical therapy and paracentesis. One month post-op, a Denver shunt was placed, tunneled through the right internal jugular vein and into the superior vena cava to the right atrial junction. At 3 months post-op, the patient was found to have biopsy-proven metastatic recurrent disease to C7 spine which was treated with stereotactic body radiotherapy and trabectedin 2.6 mg; however, by 19 months post-operation, the patient had progression of disease with metastasis to the lungs and liver and was restarted on systemic therapy with eribulin and lenvatinib. The patient has maintained normal cardiac function, with an ejection fraction of 55%–60%, and is continued to be followed both by surgical and hematology oncology.

## Discussion

Leiomyosarcoma is a sarcomatous tumor comprising 10%–20% of all sarcomas. Primary vascular leiomyosarcoma is a rare subtype that arises from smooth muscle cells located in the middle layer of the venous wall and represents 2% of all LMS (9, 10). They rarely affect the IVC, accounting for only 5% of all vascular LMS (11, 12), with fewer than 400 cases reported in the literature (10) since its first description in 1871 (13), and its first surgical resection described in 1928 (6). Approximately 50 cases of intravascular tumor spread and extension to the RA have been documented in the literature, yet the therapeutic approaches for these cases remain unclear. Wachtel et al. showed that out of 19 tumors involving the RA, 11 required the use of CPB. However, they did not provide any further surgical details about the other eight patients (12, 14).

Leiomyosarcomas may affect different segments of the IVC. Segment I is below the renal veins and is the most common site of involvement; it can cause symptoms such as abdominal pain and lower-extremity edema. Segment II is between the renal and hepatic veins and causes abdominal pain and distension; if it extends into the renal veins, it may cause renal vein thrombosis, nephrotic syndrome, or arterial hypertension. Segment III is above the hepatic veins and is the least common site of involvement. The tumor in this segment is associated with Budd-Chiari syndrome (15, 16).

These tumors require multimodal imaging for diagnosis, staging, and follow-up. However, due to the rarity of this tumor, there is a lack of large-scale studies comparing the accuracy of CT and MRI in this setting. A comprehensive MRI protocol should include T1-weighted, T2-weighted, diffusion-weighted, and contrast-enhanced sequences (17).

To date, there is no standardized definition of resectability with these types of tumors; current guidelines state that technically unresectable tumors are those involving vital structures or tumors whose removal would cause unacceptable morbidity, and patients with poor performance status are deemed medically unresectable (18). Management may require resection of more than one organ; Wachtel et al. reported that the most frequently resected organ was the right kidney, followed by the right adrenal gland and partial liver (12). Perhavec et al. found the most common reasons for technical unresectability to be the involvement of the superior mesenteric vessels, followed by the involvement of bone, portal vein, or hepatic axis (6). The effect of vascular resection on survival and complications in patients with retroperitoneal sarcoma was investigated by Hu et al., in a systematic review and meta-analysis that analyzed four studies with 959 patients for recurrence-free survival (RFS) and three studies with 284 patients for overall survival (OS). The meta-analysis showed that vascular resection did not improve RFS or OS compared with tumor resection alone. Vascular resection was also associated with a higher rate of major complications in one study, but no deaths were reported in two studies (19). When managing an intracardiac tumor thrombus, resection is preferred and intraoperative CPB should be considered to facilitate resection and maintain perfusion (20–22).

Their rarity poses a great challenge, as the benefits of systemic treatment are undefined, and surgical management remains controversial. Neoadjuvant therapy can be used to reduce tumor size and improve resectability (17). If complete resection is not feasible, debulking and radiation therapy can offer good palliation (23). Adjuvant chemotherapy consists of a combination of agents such as dacarbazine, doxorubicin, ifosfamide/cisplatin, epirubicin, or cyclophosphamide (17). However, surgical resection with a tumor-free margin seems to be the only option with the highest probability to cure this disease (24). It has been shown that radical resection of the tumor leads to improved long-term survival, with a 5-year survival rate ranging between 31% and 67% (25). A randomized clinical trial by Pautier et al. showed that patients with metastatic or unresectable leiomyosarcomas had a significant improvement in progression-free survival when they received doxorubicin and trabectedin, compared with doxorubicin alone. The combination therapy had more toxicity, but it was manageable. Therefore, doxorubicin and trabectedin could be an option for the initial treatment of metastatic leiomyosarcomas (7). Immunotherapy is an emerging therapeutic strategy for sarcomas, which poses significant challenges for treatment; however, the efficacy and safety of immunotherapy for sarcomas are limited by several factors, such as the low immunogenicity of sarcoma antigens, the immunosuppressive tumor microenvironment, and the lack of predictive biomarkers. Interferon-alpha and interleukin-2 have shown modest activity in some sarcoma subtypes but are limited by toxicity and low response rates. Newer cytokines, such as interleukin-12 and interleukin-15, are being investigated in preclinical and early clinical studies. Adoptive cell therapy, which involves the infusion of engineered or activated immune cells, such as tumor-infiltrating lymphocytes, natural killer cells, or chimeric antigen receptor (CAR) T cells, has shown promising results in preclinical models and early-phase clinical trials. Still, the challenges include the identification of specific and safe targets, optimization of cell expansion and persistence, and toxicity management. Lastly, immune checkpoint modulators like PD-1, PD-L1, and CTLA-4 have been evaluated, but the response rates have been low and variable across different sarcoma subtypes. Other monoclonal antibodies that target tumor-associated antigens, such as CD47, CD70, and GD2, are being explored in preclinical and clinical studies (26).

The prognosis of PVL is impacted by tumor size, histological grade, and location. The best survival outcomes are achieved by radical resection with negative margins, small tumor size, and no metastasis at surgery. Segment II IVC LMS has a better prognosis because it causes earlier symptoms by compressing nearby organs. Histologic grades do not affect prognosis much, as IVC leiomyosarcomas are aggressive regardless (12, 27).

In 1996, Mingoli et al. reported that most IVC LMS patients with upper-segment involvement were almost inoperable, with a median survival of only 1 month (15). In 2012, Fiore et al. reported their experience with surgical management of IVC LMS. They performed complete tumor removal and IVC resection in 15 patients. They achieved low complication rates, high graft patency rates, and an 80% survival rate at a median follow-up of 31.6 months. However, they also observed local and distant recurrence in some patients (28). In 2020, Ong et al. evaluated their surgical outcomes in 30 patients with IVC LMS. They performed en bloc resection of the tumor and the IVC. In nine patients, they also performed right nephrectomy and left renal vein ligation, without compromising renal function. They reported a low rate of postoperative complications and no surgical mortality. Most of the tumors were high-grade and had clear margins. Five patients developed local recurrence and 11 patients developed distant metastasis. The median overall survival was 41 months, and the 5-year overall survival rate was 32.1% (29).

We describe a rare case of LMS arising from the IVC and extending into the RA, posing significant challenges for systemic and surgical management. We anticipate that the number of tumors that were previously deemed unresectable will increase as surgical and oncologic therapeutics advance, allowing for more resectable cases. This case demonstrates the importance of a multidisciplinary team approach to optimize surgical outcomes and the feasibility of complex surgical resection for tumors with extensive vascular and adjacent organ involvement.

## Data availability statement

Data sharing is not applicable to this article as no datasets were generated or analyzed during the current study.

## Ethics statement

Written informed consent was obtained from the participant/patient(s) for the publication of this case report.

## Author contributions

LC: Investigation, Project administration, Writing – original draft, Writing – review & editing. MT: Data curation, Project administration, Writing – original draft, Writing – review & editing. AL: Writing – review & editing. TS: Writing – review & editing. JG: Writing – review & editing. GC: Funding acquisition, Resources, Supervision, Writing – original draft, Writing – review & editing.
